# Association between perioperative statin–ezetimibe therapy and reoperation following arthroscopic rotator cuff repair: A TriNetX database study

**DOI:** 10.1007/s40199-026-00678-y

**Published:** 2026-10-25

**Authors:** Ronak Mahatme, Anish Gangavaram, Nishanth Muthusamy, Shawn Moore, Thomas Nayak, Kasey Shao, Brian Grawe, David Bernholt

**Affiliations:** Department of Orthopaedics & Sports Medicine, University of Cincinnati, Cincinnati, USA

**Keywords:** Arthroscopic rotator cuff repair, Hyperlipidemia, Ezetimibe, Statin, Tendon healing

## Abstract

**Background:**

Hyperlipidemia (HLD) impairs tendon healing and is associated with increased revision risk following arthroscopic rotator cuff repair (ARCR). While statins may partially mitigate this effect, residual dyslipidemia may impair healing. Ezetimibe, through inhibition of intestinal cholesterol absorption, has demonstrated favorable effects on tendon healing in preclinical studies.

**Objectives:**

This study evaluated whether perioperative statin–ezetimibe therapy is associated with improved postoperative outcomes following ARCR compared with statin monotherapy and patients without HLD.

**Methods:**

A retrospective cohort study was conducted using the TriNetX Research Network. Adult patients undergoing ARCR were stratified into three cohorts based on HLD status and perioperative (± 6 months) lipid-lowering therapy: (1) documented HLD treated with statin–ezetimibe prescriptions, (2) patients without documented HLD or lipid-lowering therapy prescriptions, and (3) documented HLD treated with statin monotherapy prescriptions. Primary comparisons were predefined between cohort 1 and cohorts 2 and 3. Propensity score matching (1:1) adjusted for demographics and comorbidities. Tear size and presence of fatty atrophy and fatty infiltration were not adjusted for. Outcomes occurring within 5 years included revision rotator cuff repair (RCR), conversion to shoulder arthroplasty, and secondary shoulder arthroscopy.

**Results:**

After matching, 1,066 patients were included in cohorts 1 and 2 and 1,114 patients in cohorts 1 and 3. Compared with patients without HLD, statin–ezetimibe therapy demonstrated similar rates of revision RCR (5.4% vs. 6.4%; RR 0.853; *p* = 0.358), conversion to shoulder arthroplasty (RR 1.227; *p* = 0.470), and secondary arthroscopy (RR 0.846; *p* = 0.260). Compared with statin monotherapy, statin–ezetimibe therapy was associated with lower rates of revision RCR (5.6% vs. 8.0%; RR 0.697; *p* = 0.023) and secondary shoulder arthroscopy (7.3% vs. 9.6%; RR 0.757; *p* = 0.048), while arthroplasty rates remained similar (RR 1.261; *p* = 0.400).

**Conclusion:**

Among patients with HLD undergoing ARCR, perioperative statin–ezetimibe therapy was associated with lower reoperation rates compared with statin monotherapy and postoperative outcomes comparable to patients without HLD. These findings support further investigation of intensified lipid-lowering therapy, while accounting for tear size and fatty infiltration.

**Supplementary Information:**

The online version contains supplementary material available at https://doi.org/10.1007/s40199-026-00678-y.

## Introduction

Hyperlipidemia (HLD) affects approximately 86 million adults in the United States and is a well-established driver of systemic tissue-level pathology beyond cardiovascular disease [1, 2]. Among musculoskeletal tissues, growing evidence indicates that dyslipidemia impairs tendon healing through cholesterol-mediated oxidative stress, fatty infiltration (FI), and disruption of collagen architecture [3–5]. Rotator cuff tears (RCT), among the most common musculoskeletal conditions in adults, represent an important clinical model for studying the impact of lipid-lowering pharmacotherapy on real-world surgical outcomes [6–8]. Imaging studies have reported rotator cuff abnormalities in up to 60% of patients over 60 years of age, with full-thickness tears increasing from 10% of patients in the 5th decade to nearly 30% among those in the 7th decade [9–12]. After unsuccessful non-operative treatment, arthroscopic rotator cuff repair (ARCR) has become the standard of care. In fact, over 250,000 rotator cuff repairs are performed annually in the United States, with over 80% of these procedures being performed arthroscopically [13, 14]. While ARCR generally provides favorable clinical outcomes, retear rates remain high, ranging from 10 to 40% depending on follow-up duration. Moreover, older age, larger tear size, FI, and HLD have been consistently associated with a greater risk of rotator cuff retear [15–19]. Progressive FI and muscle atrophy after RCTs lead to tendon repair failure and poor outcomes. Hypercholesterolemia characterized by high levels of low-density lipoprotein (LDL) cholesterol is a risk factor for worsened outcomes in rotator cuff repairs, potentially due to increased inflammation, which may lead to FI and muscle atrophy [20, 21]. Animal studies have shown that certain medication may in fact suppress FI and muscle atrophy after rotator cuff tears [22].

Given the high burden of retear and the role of HLD as a modifiable risk factor, there is growing interest in lipid-lowering pharmacotherapy as a strategy to improve tendon healing outcomes [3, 23]. HLD has been associated with accelerated FI and degenerative changes, impairing rotator cuff tendon quality and structural integrity when healing [24, 25]. By improving lipid control, statin use is thought to lower FI in the healing rotator cuff tendon and promote healing by modulating proinflammatory cyclooxygenase-2 and prostaglandin E_2_ after acute tendon injury [26]. Among patients undergoing ARCRs, those with high perioperative low-density lipoprotein (LDL) and cholesterol had higher revision rates, while statin use mitigated this risk, approximating revision rates of normolipidemic patients [18]. However, even among those on statins, evidence suggests that inadequate lipid control appears to drive worse outcomes, highlighting the need for complementary lipid-lowering strategies [27]. One such strategy is complementary ezetimibe use, a common statin adjunct that blocks intestinal cholesterol absorption [28]. In a rat rotator cuff repair model, ezetimibe was evaluated in combination with atorvastatin, demonstrating less FI of the tendon, improved biomechanical strength, and enhanced collagen formation compared to controls [29].

Using the TriNetX real-world electronic health record network, a federated database comprising de-identified patient data from over 150 million individuals across more than 100 U.S. healthcare organizations, this study evaluates whether combination statin–ezetimibe therapy is associated with improved 5-year reoperation rates following ARCR in patients with HLD, compared with statin monotherapy. Outcomes are further compared with a cohort without documented HLD or lipid-lowering therapy prescriptions. We hypothesized that among patients with HLD, combination statin–ezetimibe therapy would be associated with lower reoperation rates compared with statin monotherapy, and outcomes comparable to patients without a documented diagnosis of HLD or lipid-lowering therapy prescriptions.

## Methods

### Data source

This retrospective cohort study utilized the TriNetX Research Network, accessed on March 26, 2026. TriNetX aggregates de-identified electronic medical records from over 150 million patients across 102 healthcare organizations in the United States, including diagnoses, procedures, medications, and laboratory values. The use of deidentified data renders this study exempt from institutional review board approval. TriNetX is compliant with HIPAA and certified to ISO 27001:2013 standards. Reporting for this study followed the Strengthening the Reporting of Observational Studies in Epidemiology (STROBE) reporting guideline.

### Cohort identification

Adult patients (≥ 18 years) undergoing ARCR (CPT 29827) were identified. Patients with a history of prior rotator cuff repair or shoulder arthroplasty were excluded. Acute traumatic RCTs were not excluded and were eligible for inclusion. Patients were stratified into three cohorts based on HLD status (ICD-10-CM E78.0-E78.5) and documented lipid-lowering medication prescription within a prespecified 6 months before or after the index ARCR: (1) HLD treated with statin–ezetimibe combination therapy, (2) patients without HLD and without documented lipid-lowering therapy, and (3) HLD treated with statin monotherapy. Combination therapy was defined by documented prescriptions to both a qualifying statin and ezetimibe within this perioperative window, whereas statin monotherapy was defined by a documented statin prescription without a documented ezetimibe prescription during the same window. Patients receiving ezetimibe monotherapy without documented statin exposure were excluded from the analysis because they did not meet the definition of either the combination therapy or statin monotherapy cohorts. These exposure definitions reflect documented medication prescriptions rather than confirmed medication adherence or continuous treatment. The database did not permit reliable determination of statin medication dosage, duration of therapy, adherence, or whether combination therapy was initiated before versus after the index procedure. Statins were identified using RxNorm codes 41127 (fluvastatin), 42463 (pravastatin), 36567 (simvastatin), 83367 (atorvastatin), 301542 (rosuvastatin), and 6472 (lovastatin). Ezetimibe exposure was defined as 10 mg once daily (RxNorm 341248). This perioperative window was selected based on prior literature evaluating lipid-lowering therapy in ARCR populations [18]. Primary comparisons were defined a priori between cohort 1 and cohorts 2 and 3.

### Propensity score matching

Propensity score matching was performed separately for the statin–ezetimibe combination therapy cohort versus the cohort without HLD or lipid-lowering therapy and the statin–ezetimibe combination therapy cohort versus the statin monotherapy cohort. A 1:1 greedy nearest-neighbor matching algorithm was used with a caliper width of 0.1 pooled standard deviations of the propensity score. The caliper was selected to restrict matches to patients with closely similar propensity scores and thereby reduce residual differences in measured baseline characteristics between treatment groups.

Matching variables were selected a priori based on their potential associations with rotator cuff pathology, postoperative outcomes, and lipid-related comorbidity. Age, sex, body mass index > 30 (ICD-10-CM Z68.3–Z68.4), tobacco use (ICD-10-CM Z72.0), and type 2 diabetes mellitus (ICD-10-CM E11) were included in both comparisons. Information regarding tear size, presence of muscle atrophy, and FI were not available and these risk factors were not adjusted for. For the comparison between statin–ezetimibe combination therapy and statin monotherapy, heart failure (ICD-10-CM I50), atherosclerosis (ICD-10-CM I70), and preoperative low-density lipoprotein (LDL; TNX Curated: 9002) were additionally included to account for differences in cardiovascular comorbidity and baseline lipid status that may influence treatment selection. Variables occurring after the index ARCR or representing postoperative management were not included in the propensity score model. Patients with missing data for matching variables were excluded, and no imputation was performed. Covariate balance was assessed using standardized mean differences (SMDs), with an absolute SMD < 0.10 considered indicative of adequate balance. SMDs were calculated before and after propensity score matching and are presented in Tables 1 and 2.

**Table 1:** Cohort characteristics before and after propensity score matching between Cohort 1 (HLD + Statin + EZ) and Cohort 2 (No HLD or lipid-lowering medication)

Variable	Before matching cohort 1 (*n* = 1,117)	Before matching cohort 2 (*n* = 59,205)	Std Diff	*p*-value	After matching cohort 1 (*n* = 1,066)	After matching cohort 2 (*n* = 1,066)	Std Diff	*p*-value
Age, years (mean ± SD)	64.0 ± 8.0	54.3 ± 11.1	1.0037	< 0.001	63.7 ± 7.9	63.8 ± 8.1	0.0187	0.667
Male, n (%)	686 (61.4%)	33,921 (57.3%)	0.0840	0.006	654 (61.4%)	647 (60.7%)	0.0135	0.756
Female, n (%)	430 (38.5%)	25,175 (42.5%)	0.0821	0.007	411 (38.6%)	419 (39.3%)	0.0154	0.722
BMI 30–39, n (%)	297 (26.6%)	5,532 (9.3%)	0.4610	< 0.001	264 (24.8%)	270 (25.3%)	0.0130	0.764
BMI ≥ 40, n (%)	86 (7.7%)	2,395 (4.0%)	0.1559	< 0.001	76 (7.1%)	68 (6.4%)	0.0299	0.490
Tobacco use, n (%)	92 (8.2%)	1,703 (2.9%)	0.2356	< 0.001	86 (8.1%)	78 (7.3%)	0.0282	0.516
Type 2 diabetes mellitus, n (%)	488 (43.7%)	3,913 (6.6%)	0.9453	< 0.001	442 (41.5%)	451 (42.3%)	0.0171	0.693

*BMI* body mass index, *EZ* ezetimibe, *HLD* HLD, *SD* standard deviation, *Std Diff* standardized mean difference

**Table 2:** Cohort characteristics before and after propensity score matching Cohort 1 (HLD + Statin + EZ) and Cohort 3 (HLD + only Statin)

Variable	Before matching cohort 1 (*n* = 1,117)	Before matching cohort 3 (*n* = 25,898)	Std Diff	*p*-value	After matching cohort 1 (*n* = 1,114)	After matching cohort 3 (*n* = 1,114)	Std Diff	*p*-value
Age, years (mean ± SD)	64.0 ± 8.0	62.8 ± 8.4	0.1453	< 0.001	63.9 ± 8.0	64.1 ± 7.9	0.0178	0.675
Male, n (%)	686 (61.4%)	15,197 (58.7%)	0.0558	0.069	683 (61.3%)	691 (62.0%)	0.0148	0.727
Female, n (%)	430 (38.5%)	10,689 (41.3%)	0.0567	0.065	430 (38.6%)	423 (38.0%)	0.0129	0.760
BMI 30–39, n (%)	297 (26.6%)	6,698 (25.9%)	0.0165	0.588	294 (26.4%)	281 (25.2%)	0.0267	0.529
BMI ≥ 40, n (%)	86 (7.7%)	2,464 (9.5%)	0.0648	0.042	86 (7.7%)	65 (5.8%)	0.0750	0.077
Tobacco use, n (%)	92 (8.2%)	2,211 (8.5%)	0.0109	0.724	92 (8.3%)	81 (7.3%)	0.0369	0.384
Type 2 diabetes mellitus, n (%)	488 (43.7%)	10,698 (41.3%)	0.0482	0.114	485 (43.5%)	484 (43.4%)	0.0018	0.966
Heart failure, n (%)	172 (15.4%)	2,152 (8.3%)	0.2206	< 0.001	169 (15.2%)	157 (14.1%)	0.0305	0.472
Atherosclerosis, n (%)	151 (13.5%)	1,972 (7.6%)	0.1929	< 0.001	148 (13.3%)	161 (14.5%)	0.0338	0.426
LDL, mg/dL (mean ± SD)	92.3 ± 41.8	95.3 ± 39.1	0.0735	0.026	92.3 ± 41.8	91.3 ± 36.9	0.0274	0.562

*BMI* body mass index, *EZ* ezetimibe, *HLD* Hyperlipidemia, *SD* standard deviation, *Std Diff* standardized mean difference

### Outcomes

Outcomes were assessed within 5 years following index ARCR and included revision rotator cuff repair (CPT 23410, 23412, 23420, 29827), conversion to total shoulder arthroplasty (CPT 23472), and secondary shoulder arthroscopy (CPT 29805, 29806, 29807, 29819–29828). The unit of analysis was the patient rather than the number of procedures; therefore, bilateral ARCR procedures and repeated ARCR procedures were not counted as separate observations, and each patient contributed a single qualifying index ARCR to the analysis. Because TriNetX does not reliably provide laterality for all procedure records, subsequent shoulder procedures could not consistently be confirmed as involving the ipsilateral shoulder. Therefore, procedure-based outcome events may have included procedures performed on the contralateral shoulder. Moreover, TriNetX does not reliably provide imaging-confirmed tendon integrity or structural retear data. Consequently, procedure-based outcomes were not considered direct measures of structural tendon healing.

### Statistical analysis

Given the medication exposure definition, the analysis evaluated the association between documented perioperative medication prescriptions and subsequent postoperative outcomes rather than the causal effect of initiating or maintaining combination therapy. Risk ratios (RRs) with 95% confidence intervals (CI) and absolute risk differences (ARD) were calculated. P-values were derived using chi-square or Fisher’s exact tests as appropriate, with statistical significance defined as *p* < 0.05.

## Results

### Cohort identification & matching

**Fig. 1:**
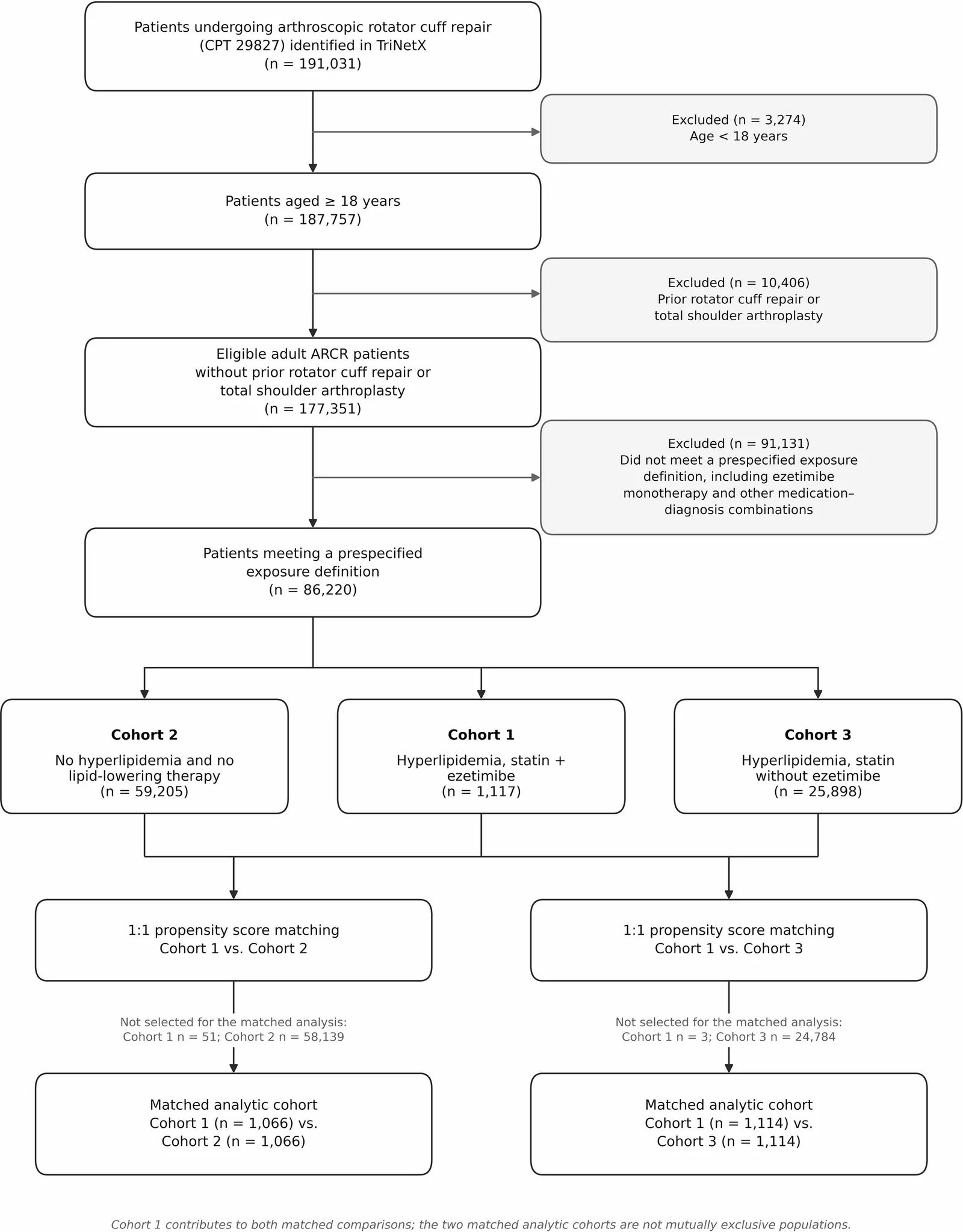
Flow diagram of patient identification, cohort construction, exclusions, and propensity score matching for patients undergoing arthroscopic rotator cuff repair. ARCR: arthroscopic rotator cuff repair

The cohort identification and exclusion process is summarized in Fig. 1. Among 191,031 patients who underwent ARCR, 187,757 were adults aged ≥ 18 years and were eligible for further cohort assessment. Patients with a history of prior rotator cuff repair or shoulder arthroplasty were excluded. Patients were subsequently stratified according to documented HLD status and perioperative lipid-lowering medication prescriptions into three prespecified cohorts: Cohort 1 (HLD treated with statin–ezetimibe), Cohort 2 (no documented HLD or lipid-lowering therapy), and Cohort 3 (HLD treated with statin monotherapy). Patients with ezetimibe monotherapy or medication exposure that did not meet the prespecified cohort definitions were excluded.

Prior to 1:1 propensity score matching of patients undergoing ARCR, Cohort 1 (HLD treated with statin–ezetimibe) included 1,117 patients, whereas Cohort 2 (no HLD or lipid-lowering therapy) comprised 59,205 patients. Following matching, both cohorts were balanced at 1,066 patients each, with adequate covariate balance achieved across the prespecified matching variables (all standardized mean differences < 0.10) (Table 1). Mean follow-up was 964 ± 663 days in the statin–ezetimibe cohort and 864 ± 696 days in the cohort without HLD or lipid-lowering therapy (*p* < 0.001), thereby corresponding to a mean difference of 100 days. Median LDL-C was 79 mg/dL (IQR, 51 mg/dL) in Cohort 1 and 103 mg/dL (IQR, 37 mg/dL) in Cohort 2 before matching, compared with 79 mg/dL (IQR, 51 mg/dL) and 96 mg/dL (IQR, 41 mg/dL), respectively, after matching. LDL-C remained incompletely balanced after matching (SMD = 0.254; *p* < 0.001).

Prior to 1:1 propensity score matching of patients undergoing ARCR, Cohort 1 (HLD + statin–ezetimibe) included 1,117 patients, whereas Cohort 3 (HLD + statin monotherapy) comprised 25,898 patients. Following matching, both cohorts were balanced at 1,114 patients each. Adequate balance was achieved for all baseline characteristics, with standardized mean differences < 0.10 for all covariates (Table 2). In the statin–ezetimibe versus statin monotherapy comparison, mean follow-up was substantially shorter in the statin–ezetimibe cohort than in the statin monotherapy cohort (960 ± 663 vs. 1,185 ± 643 days, respectively; *p* < 0.001), corresponding to a mean difference of approximately 225 days. Median LDL-C was 84 mg/dL (IQR, 54 mg/dL) in Cohort 1 and 89 mg/dL (IQR, 48 mg/dL) in Cohort 3 before matching, compared with 84 mg/dL (IQR, 54 mg/dL) and 85 mg/dL (IQR, 45 mg/dL), respectively, after matching. LDL-C was well balanced after matching (SMD, 0.027; *p* = 0.562).

### Cohort 1 (HLD + Statin + Ezetimibe) vs. Cohort 2 (No HLD or Lipid-lowering medication)

**Fig. 2:**
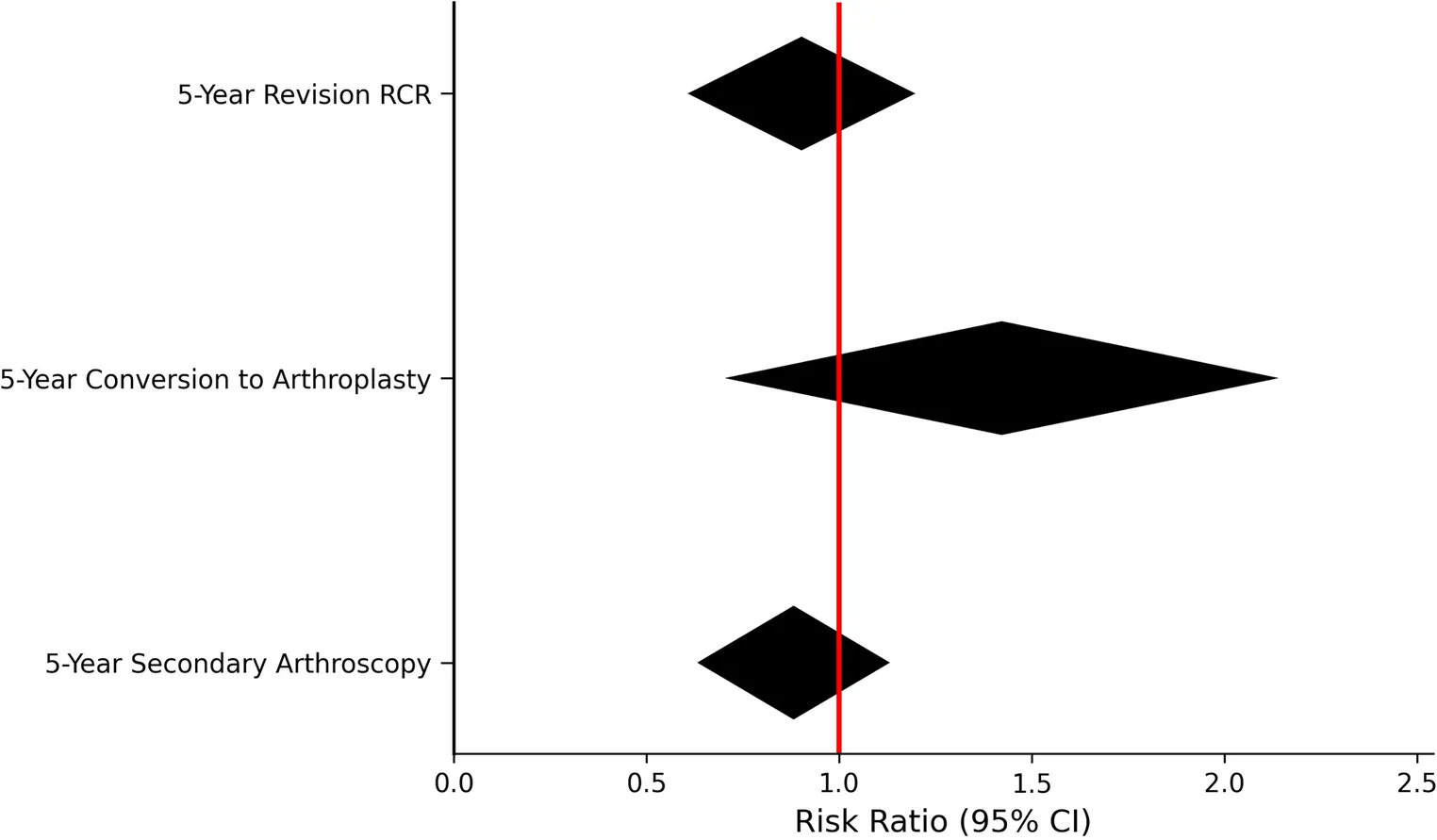
Postoperative outcomes between Cohort 1 (HLD + Statin + EZ) and Cohort 2 (No HLD or lipid-lowering medication) following ARCR. ARCR: arthroscopic rotator cuff repair; CI: confidence interval; EZ: ezetimibe; HLD: hyperlipidemia; RCR: rotator cuff repair

Within 5 years after ARCR, patients in Cohort 1 (HLD treated with statin–ezetimibe) and Cohort 2 (no HLD or lipid-lowering therapy) demonstrated similar rates of revision rotator cuff repair (5.4% vs. 6.4%; RR 0.853; 95% CI, 0.607–1.198; *p* = 0.358), conversion to shoulder arthroplasty (2.5% vs. 2.1%; RR 1.227; 95% CI, 0.703–2.141; *p* = 0.470), and secondary shoulder arthroscopy (7.2% vs. 8.5%; RR 0.846; 95% CI, 0.632–1.132; *p* = 0.260) (Table 3; Fig. 2).

**Table 3:** Postoperative outcomes between Cohort 1 (HLD + Statin + EZ) and Cohort 2 (No HLD or lipid-lowering medication) following ARCR

Variable	Cohort 1 events (Risk %) (*n* = 1,066)	Cohort 2 events (Risk %) (*n* = 1,066)	Absolute risk difference (%)	RR (95% CI)	*p*-value
5-Year Revision RCR	58 (5.4%)	68 (6.4%)	1.0	0.853 (0.607–1.198)	0.358
5-Year Conversion to Arthroplasty	27 (2.5%)	22 (2.1%)	0.4	1.227 (0.703–2.141)	0.470
5-Year Secondary Arthroscopy	77 (7.2%)	91 (8.5%)	1.3	0.846 (0.632–1.132)	0.260

*ARCR* arthroscopic rotator cuff repair, *CI* confidence interval, *EZ* ezetimibe, *HLD* hyperlipidemia, *RCR* rotator cuff repair, *RR* risk ratio

### Cohort 1 (HLD + Statin + Ezetimibe) vs. Cohort 3 (HLD + only Statin)

**Fig. 3:**
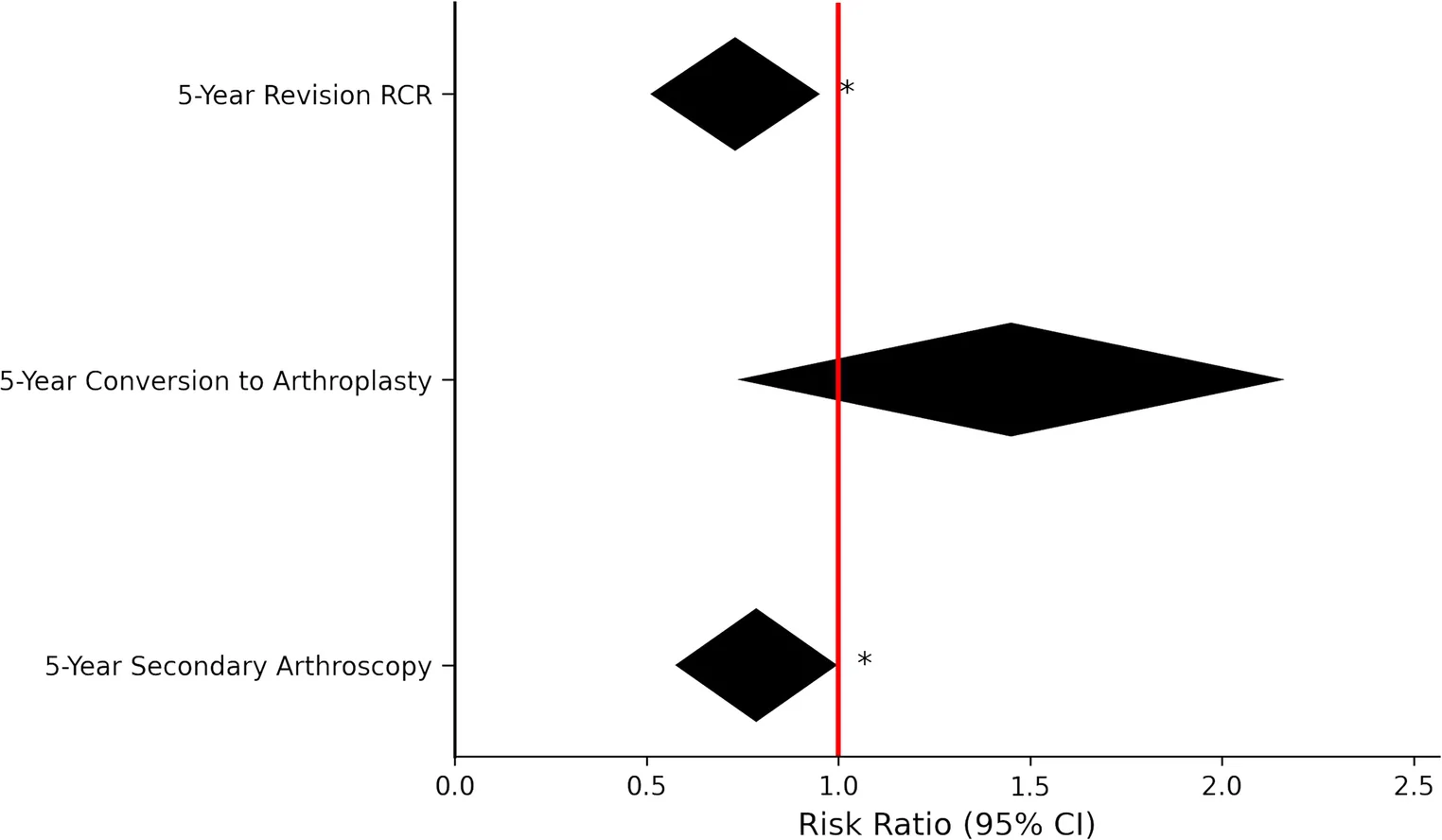
Postoperative outcomes between Cohort 1 (HLD + Statin + EZ) and Cohort 3 (HLD + only Statin) following ARCR. * indicates *p* < 0.05. ARCR: arthroscopic rotator cuff repair; CI: confidence interval; EZ: ezetimibe; HLD: hyperlipidemia; RCR: rotator cuff repair

Within 5 years after ARCR, patients in Cohort 1 (HLD treated with statin–ezetimibe) demonstrated significantly lower rates of revision rotator cuff repair compared with Cohort 3 (HLD with statin monotherapy) (5.6% vs. 8.0%; RR 0.697; 95% CI, 0.509–0.953; *p* = 0.023). Conversion to shoulder arthroplasty was similar between groups, occurring in 2.6% of Cohort 1 and 2.1% of Cohort 3, without a statistically significant difference (RR 1.261; 95% CI, 0.734–2.166; *p* = 0.400). Secondary shoulder arthroscopy was significantly lower in Cohort 1 than Cohort 3 (7.3% vs. 9.6%; RR 0.757; 95% CI, 0.574–0.998; *p* = 0.048) (Table 4; Fig. 3).

**Table 4:** Postoperative outcomes between Cohort 1 (HLD + Statin + EZ) and Cohort 3 (HLD + only Statin) following ARCR

Variable	Cohort 1 Events (Risk %) (*n* = 1,114)	Cohort 3 Events (Risk %) (*n* = 1,114)	Absolute Risk Difference (%)	RR (95% CI)	*p*-value
5-Year Revision RCR	62 (5.6%)	89 (8.0%)	2.4	0.697 (0.509–0.953)	**0.023**
5-Year Conversion to Arthroplasty	29 (2.6%)	23 (2.1%)	0.5	1.261 (0.734–2.166)	0.400
5-Year Secondary Arthroscopy	81 (7.3%)	107 (9.6%)	2.3	0.757 (0.574–0.998)	**0.048**

*ARCR* arthroscopic rotator cuff repair, *CI* confidence interval, *EZ* ezetimibe, *HLD* hyperlipidemia, *RCR* rotator cuff repair, *RR* risk ratio

Kaplan–Meier curves depicting revision-free survival for both comparisons are presented in Supplementary Figs. 1 and 2.

## Discussion

These real-world findings demonstrate an association between perioperative statin–ezetimibe prescriptions and lower rates of revision RCR and secondary shoulder arthroscopy compared with statin monotherapy. Outcomes in the combination therapy cohort were also comparable to those observed in a matched cohort without HLD or lipid-lowering therapy. However, given the observational design, potential for residual confounding, and exposure misclassification, these findings should not be interpreted as evidence that combination therapy directly improves structural tendon healing, as the present study evaluated procedure-based reoperation outcomes rather than imaging-confirmed tendon integrity.

The comparison between the statin–ezetimibe cohort and patients without documented HLD or lipid-lowering therapy warrants additional caution. Although propensity score matching improved baseline comparability, LDL-C remained incompletely balanced between these groups (SMD = 0.254). This residual imbalance may reflect underlying differences in lipid status between patients with documented HLD receiving lipid-lowering therapy and those without documented HLD or lipid-lowering therapy and represents a potential source of residual confounding. Accordingly, the observed similarity in postoperative outcomes between these groups should be interpreted as a contextual association rather than evidence of equivalence or a causal treatment effect. In contrast, LDL-C was well balanced in the statin–ezetimibe versus statin-monotherapy comparison (SMD = 0.027), supporting greater comparability for the treatment-specific analysis.

The pharmacological rationale for dual lipid-lowering therapy in this context is mechanistically grounded. Statins reduce LDL-C through hepatic HMG-CoA reductase inhibition, while ezetimibe blocks intestinal cholesterol absorption via NPC1L1, yielding complementary and additive LDL-C reductions [30]. Real-world evidence confirms that statin–ezetimibe combination therapy achieves significantly greater LDL-C target attainment than statin monotherapy, with a 29% higher probability of reaching guideline-recommended LDL-C goals in a large observational cohort [31]. At the tissue level, elevated cholesterol promotes oxidative stress and increases apoptosis and autophagy in tendon-derived stem cells, impairing the biologic environment necessary for tendon repair [32]. Additionally, cholesterol deposition within tendon tissue has been shown to alter both macrostructural and ultrastructural collagen organization, reducing tendon mechanical strength and elasticity [5]. Prior clinical studies have similarly demonstrated that HLD is associated with increased rates of rotator cuff disease, failed healing, and revision surgery following ARCR [15, 24, 25]. Recent evidence further supports this association, with a systematic review and meta-analysis reporting a significantly increased risk of rotator cuff retear among patients with dyslipidemia (OR 1.32, 95% CI 1.06–1.64) [33]. These findings provide additional clinical support for the relationship between dyslipidemia and rotator cuff repair failure and reinforce the rationale for investigating whether lipid-lowering therapy may modify postoperative outcomes.

In contrast, lipid-lowering therapies may improve the tendon healing environment through both systemic lipid reduction and direct anti-inflammatory effects. Dolkart et al. demonstrated that statins enhance rotator cuff healing through activation of the COX-2/PGE2/EP4 pathway, while Yoon et al. showed that combined ezetimibe/atorvastatin therapy in a rat rotator cuff repair model reduced FI, improved collagen organization, and increased bone–tendon interface strength [26, 29]. The present findings provide early clinical evidence supporting the potential translational relevance of these preclinical observations in human ARCR populations. Therefore, incorporating tear size, FI, and muscle atrophy into statistical analysis would help scientists to understand the role of these factors as a direct contributor to surgical failure or consequence of high HLD. This is important as these factors are often not reversible while HLD is a reversible factor.

Importantly, the results of our study expand upon prior literature evaluating statin therapy alone in rotator cuff repair populations. Cancienne et al. demonstrated that patients with perioperative total cholesterol levels greater than 240 mg/dL had significantly higher rates of revision rotator cuff surgery following ARCR compared with patients with normal cholesterol levels (18.1% vs. 1.9%; *p* < 0.001), while concomitant statin therapy reduced revision rates to levels more comparable with patients without a documented diagnosis of HLD or lipid-lowering therapy prescriptions (6.7% vs. 5.9%; *p* = 0.87) [18]. More recently, Lee et al. reported that patients with missed statin therapy and elevated 10-year atherosclerotic cardiovascular disease risk scores demonstrated significantly increased retear rates following ARCR despite statin treatment (17.9% vs. 9.9%; *p* = 0.002), further suggesting that incomplete lipid control may continue to adversely affect tendon healing [27]. A recent systematic review and meta-analysis found no significant difference in retear rates between statin users and nonusers, although statin use was associated with a modest reduction in revision surgery (RR 0.83, 95% CI 0.70–1.00) [34]. These findings suggest that the potential effects of lipid-lowering therapy may differ between structural tendon healing and clinically recognized revision surgery. In contrast, a recent retrospective observational study of 620 patients found no significant association between serum lipid levels or statin use and MRI-confirmed retear following ARCR, although older age, male sex, and large-to-massive tear size were significant predictors of retear [35]. These findings underscore the uncertainty surrounding the relationship between lipid-lowering therapy and structural rotator cuff healing, which was not directly assessed in the present study. Our study builds upon these findings by evaluating whether adjunctive ezetimibe therapy is associated with postoperative outcomes beyond those observed with statin monotherapy, with combination therapy associated with a 30.3% relative reduction in revision rotator cuff repair rates (5.6% vs. 8.0%; *p* = 0.023) and a 24.0% relative reduction in secondary shoulder arthroscopy rates (7.3% vs. 9.6%; *p* = 0.048) within 5 years postoperatively. Given that ezetimibe and statins act through complementary mechanisms of cholesterol reduction, the observed association may be related to differences in lipid control between treatment groups; however, postoperative lipid measurements were unavailable to directly evaluate this hypothesis.

From a pharmacotherapeutic perspective, these findings are noteworthy because both statins and ezetimibe are widely available, well-established lipid-lowering agents routinely prescribed in patients with hyperlipidemia. Although lipid management is traditionally pursued to reduce cardiovascular risk, the present results suggest that perioperative lipid-lowering therapy may be associated with musculoskeletal outcomes following ARCR [3, 30]. If validated in prospective studies, combination statin–ezetimibe therapy warrants further investigation as a potentially modifiable pharmacologic factor associated with postoperative outcomes following ARCR.

Despite these findings, the effects of statins on tendon health remain somewhat conflicting within the broader literature. Prior investigations have reported associations between statin therapy and increased rates of tendinopathy and tendon rupture, although these effects may vary based on tendon type and statin exposure level [36]. More recent evidence suggests that the increased risk associated with statin therapy may be tendon-specific, with elevated rates observed in conditions such as trigger finger, radial styloid tenosynovitis, and Achilles tendinopathy, while potentially demonstrating protective effects in rotator cuff disease [37]. Taken together, our findings suggest that within the setting of ARCR, combination statin–ezetimibe therapy was associated with more favorable postoperative reoperation outcomes in this ARCR population. However, additional prospective clinical and histologic studies remain necessary to better characterize the biologic effects of optimizing lipid-lowering therapy on tendon tissue healing and remodeling following ARCR [38–40].

### Limitations

This study has several limitations inherent to retrospective database investigations. First, the TriNetX database relies on accurate coding practices and does not provide granular clinical information regarding tear size, tear chronicity, tendon retraction, Goutallier FI, muscle atrophy, tendon quality, or imaging-confirmed healing. The database also does not reliably distinguish acute traumatic from degenerative tear etiology, which may contribute to clinical heterogeneity within the study population. These factors are important determinants of rotator cuff healing and revision risk and could differ between medication exposure groups. Similarly, procedural variables including repair construct, number and type of anchors, surgical technique, surgeon experience, and intraoperative assessment of tissue quality were unavailable. Postoperative rehabilitation protocols, adherence to rehabilitation, and patient compliance were also not captured. Consequently, substantial residual confounding may remain despite propensity score matching, as matching can only account for measured variables available within the database. LDL-C also remained incompletely balanced between the statin–ezetimibe cohort and the cohort without documented HLD or lipid-lowering therapy after matching (SMD = 0.254). This residual imbalance may reflect underlying differences in lipid status between these clinically distinct populations and represents a potential source of residual confounding when interpreting the comparison with patients without HLD. In contrast, LDL-C was well balanced between the statin–ezetimibe and statin-monotherapy cohorts after matching (SMD = 0.027), supporting greater comparability for the treatment comparison.

Second, medication exposure was defined using documented statin and ezetimibe prescriptions within a ± 6-month perioperative window and therefore does not establish medication adherence, treatment duration, dosage, or continuity of therapy. In addition, the available data did not permit reliable determination of whether combination therapy was initiated before or after the index ARCR. Although ezetimibe exposure was defined using the standard 10-mg once-daily regimen, the substantial variability in statin prescriptions within TriNetX did not permit reliable classification of statin intensity or consistent comparison of dose equivalents across cohorts. Consequently, differences in statin intensity and cumulative lipid-lowering exposure could not be fully accounted for and may represent sources of residual confounding. Moreover, patients classified as receiving combination therapy may have differed in the timing and duration of exposure, and medication records may not reflect actual medication use throughout the postoperative healing period. These factors introduce potential exposure misclassification and limit interpretation of the observed association as a treatment effect.

Third, postoperative lipid values and longitudinal measures of LDL reduction were unavailable, limiting our ability to determine whether the observed associations were related to the degree or duration of lipid control. The database also cannot distinguish structural tendon retears detected on imaging from clinically recognized failures that result in revision surgery. In addition, TriNetX does not reliably provide laterality for all procedure records, preventing consistent distinction between ipsilateral procedures related to the index ARCR and procedures performed on the contralateral shoulder. Consequently, some revision RCR or secondary shoulder arthroscopy events may have been unrelated to the index repair, resulting in potential outcome misclassification. Accordingly, these procedure-based outcomes should be interpreted as healthcare utilization-based proxies for postoperative failure rather than direct measures of tendon integrity.

An additional limitation is variable longitudinal follow-up inherent to the TriNetX electronic health record network. Although outcomes were evaluated within a prespecified 5-year postoperative window, individual patients did not necessarily have continuous observation throughout the entire period. Importantly, follow-up duration was substantially shorter in the statin–ezetimibe cohort than in the statin monotherapy cohort (960 ± 663 vs. 1,185 ± 643 days, respectively; *p* < 0.001), representing a mean difference of approximately 225 days. Because longer observation provides greater opportunity for postoperative events to be captured, patients in the statin monotherapy cohort had greater opportunity for revision rotator cuff repair, secondary shoulder arthroscopy, and other postoperative procedures to be recorded. This difference in available follow-up may therefore have contributed to differential outcome ascertainment and could bias the observed comparison toward lower cumulative event rates in the statin–ezetimibe cohort. TriNetX did not provide a patient-level measure of continuous observation throughout the 5-year period that would permit calculation of a conventional loss-to-follow-up or censoring rate in this analysis. Therefore, the reported 5-year event estimates should be interpreted as cumulative events captured within the available observation period rather than as estimates based on complete 5-year follow-up for every patient. Because patient-level continuous observation and censoring could not be reliably characterized, sensitivity analyses specifically accounting for differential follow-up duration were not feasible without making assumptions regarding the completeness and timing of follow-up. Kaplan–Meier curves were therefore used to provide a graphical representation of revision-free survival over time, while formal outcome comparisons were based on cumulative events captured within the available 5-year observation window.

Finally, despite adjustment for measured baseline characteristics, selection bias and differences in healthcare utilization, prescribing patterns, disease severity, and treatment intensity cannot be excluded. Patients receiving combination therapy may differ from those receiving statin monotherapy in disease severity, healthcare utilization, physician prescribing patterns, cardiovascular risk, treatment intensity, or other factors not captured in the database. Given these limitations, the findings should be considered hypothesis-generating and interpreted as associations rather than causal treatment effects. Prospective studies incorporating serial lipid measurements, medication adherence, detailed tear characteristics, imaging-confirmed healing, and standardized rehabilitation protocols are warranted to determine whether intensified lipid-lowering therapy directly influences rotator cuff healing.

## Conclusion

In this large real-world study, perioperative statin–ezetimibe combination therapy was associated with lower rates of revision rotator cuff repair and secondary shoulder arthroscopy at 5 years compared with statin monotherapy, with similar postoperative outcomes to patients without HLD or lipid-lowering therapy. These findings support further investigation of statin–ezetimibe combination therapy as a potentially modifiable factor in postoperative outcomes following ARCR. Prospective studies incorporating direct lipid monitoring, medication adherence, preoperative tear size and fatty infiltration, and imaging-confirmed healing endpoints are warranted to further investigate the biological mechanisms and clinical role of combined lipid-lowering therapy in tendon healing.

## Supplementary Information

Below is the link to the electronic supplementary material.

Supplementary Material 1

Supplementary Material 2

## Data Availability

Data supporting the findings are available through the TriNetX Research Network and are subject to institutional licensing restrictions. Aggregate analytic outputs are available from the corresponding author upon reasonable request.
